# Exclusion From Social Relations and Loneliness: Individual and Country-Level Changes

**DOI:** 10.1093/geroni/igaa057.2509

**Published:** 2020-12-16

**Authors:** Marja Aartsen, Deborah Morgan, Lena Dahlberg, Charles Waldegrave, Sarmitė Mikulionienė, Gražina Rapolienė, Giovanni Lamura

**Affiliations:** 1 OsloMet Oslo Metropolitan University, Oslo, Norway; 2 Swansea University Wales, Swansea, Wales, United Kingdom; 3 Aging Research Center, Karolinska Institutet, Solna, Stockholms Lan, Sweden; 4 Family Centre Social Policy Research Unit, Lower Hutt, Wellington, New Zealand; 5 Lithuanian Social Research Centre, Vilnius, Vilniaus Apskritis, Lithuania; 6 Centre for Socio-Economic Research on Ageing, Ancona, Marche, Italy

## Abstract

Social isolation and loneliness have profound implications for quality of life and health and welfare budgets, but interventions to reduce loneliness are limited effective. The aim of this study is to examine the often-ignored impact of macro-level drivers of loneliness, in addition to micro-level drivers by adopting a cross-national perspective. We use longitudinal data from 2013 and 2015 from the Survey of Health, Aging, and Retirement in Europe (SHARE), combined with macro-level data from additional sources. Our study confirms that key micro-level drivers of loneliness are gender, health and partnership status, frequency of contact with children and changes therein. Macro level drivers are level of safety in the neighbourhood, and poverty and social deprivation of a society. In order to understand and reduce loneliness we require not just a focus on individual risk factors, behaviours and expectations, but also on macro-level factors that are associated with exclusion from social relations.

